# Case Report of Leprosy in Central Florida, USA, 2022

**DOI:** 10.3201/eid3101.231370

**Published:** 2025-01

**Authors:** Austin B. Auyeung, Saphra Sohail, Marie Kima

**Affiliations:** Author affiliation: HCA North Florida Hospital, Gainesville, Florida, USA

**Keywords:** leprosy, Hansen disease, *Mycobacterium leprae*, PCR, bacteria, Florida, United States

**To the Editor:** We read with interest about the leprosy case in central Florida, USA, described by Bhukhan et al. (*1*). We report a similar case of leprosy (also known as Hansen disease), diagnosed in a 55-year-old female patient in northern Florida, that exhibited tuberculoid features. *Mycobacterium leprae* was detected by PCR in multiple biopsied lesions, confirming the diagnosis.

**Figure 1:**
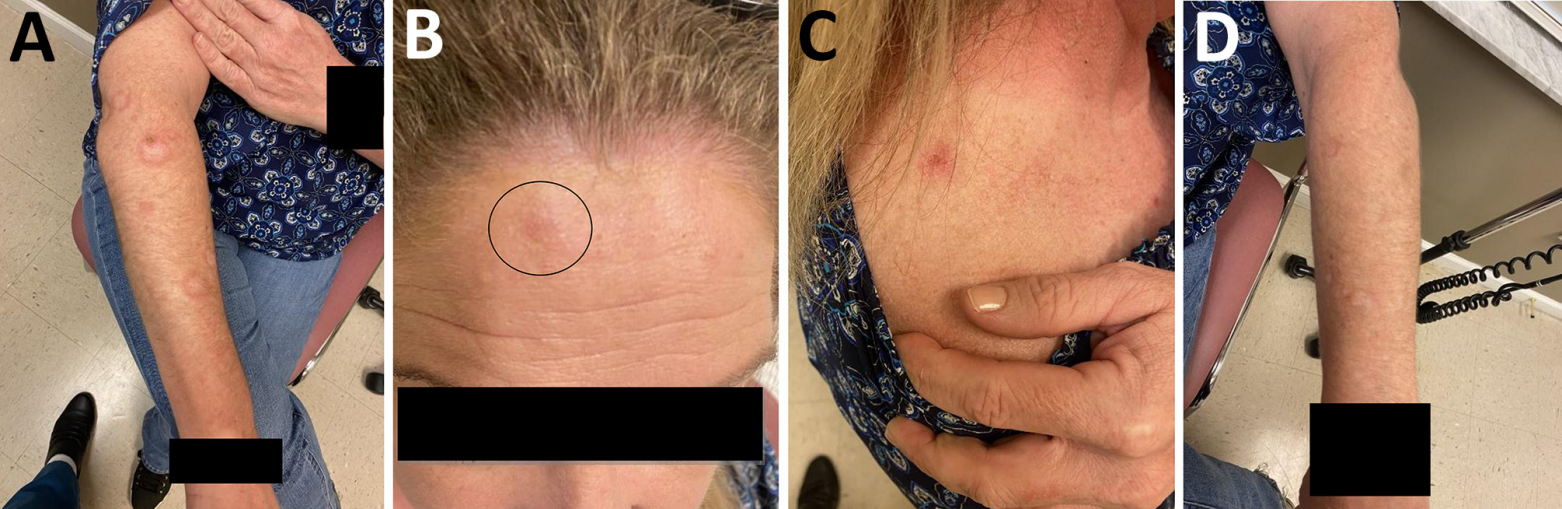
Leprosy lesions in a 55-year-old female patient in north Florida, USA. Multiple hypopigmented plaques with erythematous borders appeared along the right posterior forearm (A), right forehead (B), right trapezius (C), and left posterior forearm (D).

**Figure 2:**
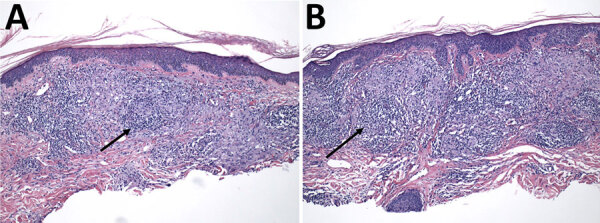
Histologic analysis of skin biopsies from a 55-year-old female patient with leprosy in north Florida, USA. Skin biopsies from right proximal ventral forearm (A) and left distal dorsal forearm (B) underwent hematoxylin and eosin staining. Arrows indicate areas of dermal granulomatous inflammation. Original magnification ×100.

The patient manifested multiple macules and patches with central clearance and erythematous borders without hypoesthesias on the right arm and shoulder (Figures 1, 2). She denied having fever, chills, or abdominal pain but reported right knee pain and swelling, suggestive of arthritis, which is not uncommon in patients with leprosy. We prescribed monthly doses of 600 mg rifampin, 400 mg moxifloxacin, and 100 mg minocycline. We added methotrexate and low-dose prednisone to the patient’s regimen to treat new neuropathy of the hands and possible leprosy reactions, according to recommendations from the National Hansen’s Disease Program. After >1 year of treatment, she remains on methotrexate, moxifloxacin, rifampin, and minocycline. Her lesions have resolved except for 1 on her right forearm, which also appears to be improving.

Contact with armadillos (*2*), the Eurasian red squirrel (*3*), and amoebae in soil (*4*) have been linked to leprosy. This patient previously lived in a house with a tree rat infestation in the attic, but it is unknown if tree rats carry leprosy. The patient works in finance and denies participating in any outdoor occupational or recreational activities. She did not report travel to a leprosy-endemic area; exposure to soil, armadillos, or squirrels; contact with someone who had been to a disease-endemic area; or contact with a person who had a confirmed case of leprosy. Because some patients with leprosy do not report traditional risk factors, it is possible that other exposure sources or zoonotic reservoirs are yet to be discovered.
